# Supplementary material for: Epidemiology and pathological progression of erythematous lip lesions in captive sun bears (Helarctos malayanus)
Source: PLoS One. 2020 Dec 1;15(12):e0243180. doi: 10.1371/journal.pone.0243180 (PMC7707555; doi:10.1371/journal.pone.0243180)
Supplement: Attachment — Submitted filename: Officer Lip lesions in sun bears Response to Reviewers.docx [file pone.0243180.s002.docx]

Manuscript ID: PONE-D-20-19581

Response to reviewers

Dear Editor

Many thanks for providing us with the opportunity to submit a revised draft of the manuscript “Epidemiology and pathological progression of erythematous lip lesions in captive sun bears (*Helarctos malayanus*)” for publication in PLoS ONE. We are grateful for the comments and feedback provided by the reviewers, and have made changes to our manuscript in response, shown as track changes in the attached manuscript draft. Please see below for a point-by-point response (in blue) to the reviewers’ comments. All line numbers refer to the revised manuscript file with tracked changes.

**Review comments to the author:**

**Reviewer #1**

1. The manuscript is well written and structured and the detailed description of the results highlights the puzzling outcome of the study, clearly urging further investigations. The authors discuss their findings with enough caution not to over interpret their sometimes contradicting findings.
   Clearly the content of the study is of importance for the scientific community working in the field of zoo medicine and animal conservation and ideally should prompt investigations in a larger set of animals.

**Author response:** Thank you for recognising the importance of this preliminary work to the field of zoo medicine and animal conservation

2. Just out of personal interest and acknowledging that Giemsa staining was also performed on histologic sections – did the authors consider Treponema sp. as a possible cause, which sometimes do not easily show in Giemsa? Somehow the sun bear lesions reminded me of Treponema cuniculi lesions in rabbits…

**Author response:**

Thank you for your comment. Whilst this is an interesting suggestion, to the authors knowledge, Treponema infection has not been recorded in Ursidae. Also, in the cohort examined here, lesions were largely restricted to erythematous lip lesions (with one vulval lesion) and no lesions were identified in other locations typically affected in cases of Treponema involvement (also in rabbits), such as the anus, nostrils and eyelids. The transition of treponema lesions to neoplasia would not be expected either, an in light of this, the authors did not consider Treponema involvement a likely differential diagnosis.

**Reviewer #2**

1. This is an informative, well-written, and sound descriptive study of herpesvirus-associated lip lesions potential squamous cell carcinoma in sun bears. This has been an emerging issue in these animals in captivity, so it warrants attention.

**Author response:** Thank you for acknowledging the importance of this issue to captive wildlife.

2. Line 381 regarding inconsistent results with pcr- (this is a comment not a critique- you might want to try laser-dissection capture microscopy of selected foci or cell types or areas of lesions most likely to have virus. This can often help with detection)-
   Immunohistochemistry may be useful in these cases as well (see the work done on otarid herpesvirus I-it cross reacted with EBV monoclonal antibodies on IHC)
   EM also can improve detection. So, I also recommend you mention IHC and EM as ancillary diagnostics that may be helpful.

**Author response:**
Thank you for your helpful comments. Immunohistochemistry is mentioned in the manuscript (see line 420) and we will consider this and your other suggestions for future work.

3. Additionally, you may want to develop a qPCR method which can be more sensitive (you mention sensitivity here already). Further,

**Author response:**

Thank you for raising this additional point regarding the potential usefulness of developing a qPCR method for increased sensitivity. In the original manuscript we mentioned this further down (line 421) in reference to detecting virus in various tissues. We have now also mentioned this in the paragraph about the discordant results. Thus, lines 389 – 393 in the revised manuscript now read:

“This is evidenced by the discordant UrHV-1 PCR results for four sun bears that had duplicate lip lesion biopsies taken on the same day and tested separately, and the relatively low proportion (59.1%) of UrHV-1 positive lip lesion biopsies from sun bears that were positive on any sample. Development of a real-time PCR assay to improve sensitivity may increase virus detection rates.”

4. In terms of the comment “Similarly, it was surprising that two cases had lip SCC in 2011 but biopsies in 2015 did not show neoplasia”, was surgical excision done of the site ? If so, it might have impacted the diagnosis.

**Author response:**

Thank you for pointing out this need for clarification. Surgical excision was not carried out at the site in 2011. We have updated the manuscript to reflect that a punch biopsy only was performed in 2011.

Please see lines 395 – 398 in the revised manuscript which now read:

“Similarly, it was surprising that two cases had lip SCC when punch biopsied in 2011 but biopsies in 2015 did not show neoplasia, possibly also reflecting the insensitivity of the biopsy sampling and virus detection methods used.”

5. Has there been any evidence of metastases in any of these cases?

**Author response:**

Thank you for this query. In general, we have not seen any obvious evidence of metastases in these cases, apart from one, which we mention in Lines 277 – 280:

“In one case (CR080), neoplastic epithelial cells were present in all sections of the tongue, and neoplastic islands did not appear to arise from or correspond with the surface epithelium, possibly indicating lingual metastases, although a primary lingual SCC could not be ruled out (Fig 2: D).”

6. line 404-4-5 please also reference work and mention otarine herpesvirus associated with genital lesions/tumors in California sea lions and add.

**Author response:**

Thank you for pointing out this omission. The manuscript has been updated to include this, with reference to King et al.’s 2002 paper “Otarine herpesvirus-1: a novel gammaherpesvirus associated with urogenital carcinoma in California sea lions (*Zalophus californianus*)” [55].

Please see lines 413 – 417 in the updated manuscript which now read:

“Although potentially a result of salivary contamination, the detection of UrHV-1 in vulval lesions was interesting given gammaherpesviruses have been found in association with genital lesions in other species including Eastern grey kangaroos (*Macropus giganteus*)[53], bottle-nosed dolphins (*Tursiops truncates*) [54], and California sea lions (*Zalophus californianus*) [55].”

**Additional changes to the manuscript**

Please note the authors have made the following additional changes (all tracked in the revised manuscript):

1. Change of virus name from Ursid herpesvirus 1 to the more correct Ursid gammaherpesvirus 1 throughout the manuscript. The abbreviation UrHV-1 remains unchanged.
2. Addition of a new current address for author Philippe Dussart
3. Addition of a statement regarding the animal ethics to the start of the Methods section, as requested by PLOS ONE in-house on re-submission

Yours sincerely

Kirsty Officer
